# Feasibility and Psychometrics of a New Test Battery for Diagnosing Patients With Schizophrenia Spectrum Disorders

**DOI:** 10.7759/cureus.107130

**Published:** 2026-04-15

**Authors:** Palak A Fichadia, Hotaik Sung, Sreya Kongala, Daria Weber, Dongliang Wang, Luba Leontieva

**Affiliations:** 1 Psychiatry, Icahn School of Medicine at Mount Sinai, New York, USA; 2 Psychiatry, Creighton University School of Medicine, Omaha, USA; 3 Psychiatry and Behavioral Sciences, Osmania Medical College, Hyderabad, IND; 4 Psychology, State University of New York Upstate Medical University, Syracuse, USA; 5 Public Health and Preventive Medicine, State University of New York Upstate Medical University, Syracuse, USA; 6 Psychiatry, State University of New York Upstate Medical University, Syracuse, USA

**Keywords:** diagnosis, mental cognition, panss scale, pictogram test, schizophrenia

## Abstract

Introduction

Schizophrenia is a complex psychiatric disorder that is often difficult to distinguish from other psychiatric conditions, particularly in acute settings. Thought disorder and concreteness are considered distinguishing cognitive features of schizophrenia. The objective of this study was to examine the feasibility and diagnostic accuracy of a brief psychological test battery in differentiating patients with schizophrenia spectrum conditions (SSC) from control patients (C) without schizophrenia. The battery combined the Positive and Negative Syndrome Scale - Negative Subscale (PANSS-N5), the Mood Differentiation Questionnaire (MDQ), and the Pictogram Test (PT).

Methods

Forty patients were recruited, including 20 patients diagnosed with schizophrenia spectrum conditions (SSC) and 20 control patients (C) without schizophrenia. Participants were administered three instruments: the Positive and Negative Syndrome Scale - Negative Subscale (PANSS-N5), the Mood Differentiation Questionnaire (MDQ), and the Pictogram Test (PT). Tests were administered by medical school graduates following structured training and were scored by professionals with psychology and medical degrees. Group differences were analyzed using independent samples statistical testing. Receiver operating characteristic (ROC) curve analysis was conducted, and the area under the curve (AUC) was calculated to evaluate the classification accuracy of each instrument in distinguishing SSC from C groups. Statistical significance was set at p < 0.05.

Results

The Mood Differentiation Questionnaire (MDQ) did not significantly differentiate between the SSC and C groups (p > 0.05; AUC = 0.652). In contrast, both the Pictogram Test (PT) (p < 0.05; AUC = 0.761) and the Positive and Negative Syndrome Scale - Negative Subscale (PANSS-N5) (p = 0.008; AUC = 0.754) significantly discriminated between SSC and C groups. The ROC analysis demonstrated acceptable to strong classification accuracy for the PT and PANSS-N5.

Conclusions

This pilot study suggests that a brief psychological test battery may have potential utility in differentiating schizophrenia spectrum conditions (SSC) from non-schizophrenia psychiatric controls following brief training. However, given the small sample size and exploratory nature of the analyses, including ROC and multivariable modeling, these findings should be interpreted with caution. While preliminary results indicate moderate discriminatory performance for the Pictogram Test and PANSS-N5, further validation in larger and more diverse samples is required. Additionally, feasibility was supported by successful administration in a clinical setting, although more objective implementation metrics are needed to substantiate this finding.

## Introduction

The Diagnostic and Statistical Manual, Fifth Edition, Text Revision (DSM-5-TR) criteria for diagnosing schizophrenia include disorganized speech [1]. Speech is a reflection of the thought process and is tightly connected to formal thought disorder (FTD). As a symptom domain, FTD appears to have a stronger genetic basis than other domains and is highly pathognomonic for psychosis. Moreover, disorganized speech can be assessed without the need for a self- or observed-based assessment of “mental state” [2]. In this context, it differs from delusions, hallucinations, anhedonia, apathy, etc., that rely on self-reports of mental state or an observer’s empathetic ability to understand another person’s mental phenomenon. Proverbs and verbal abstraction have long been connected in the minds of psychiatrists, as this highlights the ability to divulge in a complex range of neurological functions, highlighting that this connection may be used as a means to test one’s neurological function more intricately [2].

According to earlier research by Hahn et al. [3], difficulty with abstract thinking, especially maladaptive, concrete thinking, is seen as a key hallmark of schizophrenia. Gorham [4] demonstrated that individuals with schizophrenia had a serious impairment in their ability to think abstractly, as seen by a significant reduction in their abstract scores and a rise in their concrete scores between controls and patients [5]. Testing abstract thinking is crucial for both diagnosing schizophrenia and tracking improvement in response to therapy [6]. Yet, there are not many specific tests that can tap into the evaluation of thinking processes.

In clinical and research settings, the most widely used technique for evaluating symptoms of schizophrenia is the Positive and Negative Syndrome Scale (PANSS) [6,7]. The Positive Syndrome Scale assesses delusions, hallucinations, and disordered thinking; the Negative Syndrome Scale assesses apathy and avolition. Together, these two scales comprise PANSS. PANSS Negative Scale Item 5 (N5) is assessed with 32 items: 16 Proverbs and 16 Similarities, which were all asked within the same time frame. The cultural background of the interviewers and their own interpretation of Similarities and Proverbs play a role in the interpretation and scoring. A new PANSS-N5 scoring guide has been developed to solve this issue [6,7].

According to Rosen et al. [8], there is a correlation between schizophrenia and organic involvement when it comes to the impairment of the abstracting function.

According to a literature search performed by Felsenheimer and Rapp [9], when patients with schizophrenia were asked to interpret Proverbs, they provided significantly more abstract interpretations (more vague) as compared to patients in the control group. In addition, the responses of patients with schizophrenia were scored higher in concreteness and bizarreness of Proverb interpretation. Understanding Proverbs appears to be more accurately described as a spectrum of abstraction and peculiar or distinctive thought [9].

Gorham (1956) [4] published the Proverbs Test, which had three parallel versions of a clinical test with an objective scoring system that included standards for both normal people and patients with schizophrenia. His test was administered in groups and scored clerically using a multiple-choice format. The purpose of that study was to assess the hypothesis that there is a decline in abstracting function from normal to chronic schizophrenia to organic patients using the Proverbs Test [10]. This test takes, in general, 20-30 minutes to administer, and scoring takes 10-15 minutes. This test requires the following qualifications of an examiner: at least a master’s degree in psychology, clinical psychology, or related fields, experience in psychological testing, and understanding of abstract thinking [10].

“Concrete” thinking is defined as the antithesis of, or the incapacity to do, abstract thinking. The study put forth by Elmore and Gorham (1957) [10] supported this theory by demonstrating that the abstract score falls as the concrete score rises. These two scores illustrate two facets of the same cognitive process, verbal abstraction. Given the score variance across the research subjects, it is plausible that people with schizophrenia exhibit more concrete thinking [10].

One of the first batteries of tests created specifically for evaluating executive functioning in both adults and children is the Delis-Kaplan Executive Function System (D-KEFS). The creation of the D-KEFS measures has the primary goal of giving psychologists a thorough instrument for evaluating a broad range of executive functions, such as inhibition, multitasking, problem-solving, cognitive flexibility, and verbal and nonverbal creativity [11].

D-KEFS takes the following qualifications of the examiner to administer and score: the examiner must have clinical training in neuropsychology (PhD) and have experience with neuropsychological assessment. It takes 90 minutes to 3 hours to administer, and scoring takes around 1-2 hours. The D-KEFS was designed to offer measures that evaluate (a) higher-level and fundamental skills to determine whether a patient’s poor performance on a task is caused by a deficit in executive function or a more basic cognitive skill impairment, and (b) strategies and error types to more accurately characterize the type of executive dysfunction [11]. According to a stated goal of the D-KEFS, multiple investigations have shown that the D-KEFS is sensitive to executive function impairments and frontal lobe dysfunction linked to a range of neurological, developmental, and psychiatric diseases [11].

Luria’s approach to neuropsychology states that sophisticated behavioral processes are dispersed throughout the brain in “functional systems,” rather than being “localized.” Therefore, for a complicated behavioral act to be done precisely and smoothly, the coordinated activity of all cortical areas responsible for the aspects of the act is required [12-14].

Luria performed the testing in four stages. The first stage consisted of gathering information regarding the patient’s history of neurological condition, current level of functioning, and their chief complaints. In the second stage, a preliminary investigation is done to assess the motor, intellectual, and linguistic deficits, after which, in the third stage, a selective investigation is done where areas showing deficits in the previous stages are further delineated by questioning and tests, assessing the highest level of functioning, such as concept formation. Finally, the fourth stage involves reaching a neuropsychological conclusion regarding the patient’s injury and deficit [12].

Vygotsky developed a concept formation assessment where the participant is given 32 blocks that vary in height, width, color, and form [13]. They are required to group the blocks into groups based on codes that are inscribed on the bottoms of the blocks. Blocks that have the same code belong to the same category; color and shape are irrelevant. One code is used for all tall and wide blocks, another for short and wide blocks, a third for tall and thin blocks, and a fourth for short and thin blocks. The respondent is not informed of the meaning of the codes. Based on the grouping and number of blocks correctly grouped in categories, a score was provided, which assessed concept formation [13]. Vygotsky blocks require the following examiner qualification: background in developmental psychology and be trained in Vygotskian theory and assessment method to administer and score for 30-60 minutes; it takes 10-15 minutes [13].

These neuropsychological assessments can be performed only by a neuropsychologist, who may not always be readily available. Additionally, these tests have fallen out of favor due to their limitations and the evolution in our understanding of various intellectual complexities [14].

This brings our attention to the need for more contemporary test batteries that can be easily administered without the prerequisite of a psychologist to administer the test. This would make reaching a diagnostic endpoint more achievable, with minimal training and within a shorter period.

We looked for additional measures that could be used in conjunction with PANSS-N5 to increase the test’s incremental validity because there are not many specific measures of the thought process that can be carried out by a wider range of mental health professionals with the appropriate training (such as medical students, psychiatry residents, and psychiatrists). When Powell (2017) [15] created the five Mood Differentiation questions, he saw that patients suffering from schizophrenia had extremely particular answers. These five questions focus on feelings that are typically universal across cultures, such as hunger, anger, and anxiety [15]. Regarding the patient’s ego organization, the responses to these questions may indicate whether they are normal/neurotic, borderline, or psychotic/pre-psychotic, which correlate to well-differentiated, poorly differentiated, and undifferentiated moods, respectively [15].

People who have trouble differentiating their moods may react in a literal or functional way. Providing a “concrete response,” for instance, would involve the patient connecting their mood to recent perceptions or concrete examples. Individuals who exhibit a lack of emotional differentiation may give a response that needs to be more accurate or more responsive when asked how they determine the presence of a certain mood. Overall, this brief examination has the potential to distinguish between people who have schizophrenia and those who do not, all the while providing insight into the patient’s thought patterns [15]. The Mood Differentiation Questionnaire requires familiarity with it from Powell’s book “Differentiation of Moods.” It takes approximately 15 minutes to administer and 10 minutes to score per patient. The inter-rater reliability and feasibility of the Mood Differentiation Questionnaire were evaluated in patients with schizophrenia spectrum disorders and non-schizophrenia patients, which demonstrated that patients with schizophrenia spectrum disorders tend to have poorly differentiated moods compared to non-schizophrenia patients. Furthermore, the Mood Differentiation Questionnaire and the accompanying scoring guide achieved strong inter-rater reliability between two scorers, who were of different cultural and educational backgrounds. With its ease of use, the Mood Differentiation Questionnaire could be easily adapted by a broad group of mental health providers to improve the accuracy of diagnosing disturbances in thinking for patients with schizophrenia [16].

The Pictogram Test (PT) is an additional evaluation tool that assesses abstract thinking and makes a distinction between people who have cognitive disorders (like schizophrenia) and those who do not [17]. It can be administered by a mental health professional, including a psychologist or a psychiatrist. In the context of assessing thought disorders in individuals with schizophrenia, this test has been shown to have strong cross-cultural applicability and inter-rater reliability in the past [17,18].

The objective of the study was to evaluate the feasibility and accuracy of a novel psychological test battery, which combined the Positive and Negative Syndrome Scale - Negative Subscale (PANSS-N5), the Mood Differentiation Questionnaire (MDQ), and the Pictogram Test, in discriminating patients with schizophrenia from those without schizophrenia.

Therefore, by integrating these evaluations (Mood Differentiation Questionnaire, PANSS-N5, and Pictogram Test), we hope that the new test battery will show the diagnostic capacity of each instrument and the combination of them for differentiating patients with schizophrenia from patients without schizophrenia.

## Materials and methods

A total of 40 patients were recruited from the State University of New York (SUNY) Upstate Medical University Hospital for this observational study using randomized stratified sampling: 20 patients with a schizophrenia spectrum diagnosis and 20 patients without a schizophrenia spectrum diagnosis. The data collection started at the end of August 2023 and was completed at the end of December 2023. All study participants were in an acute, inpatient, psychiatric, 24-bed unit of an academic hospital in central New York, USA. The unit admits patients from the emergency department, medical floors, and outlying hospitals. The study was approved by the institutional review board (IRB) with ethical approval number #2004117, and was per Helsinki Declaration requirements. The patients suitable for participation in the study were identified by the attendings in the morning; they should have a diagnosis of schizophrenia or be part of the schizophrenia spectrum disorders, be of legal age, have no physical comorbidities, and have a mixed cultural background for diversity. We did not approach patients who were medically compromised, had a dementing illness, or had a documented intellectual disability. The review of the chart verified the patient’s diagnosis. The patients were approached for study participation with the consent form, and once they agreed and signed the consent, they were enrolled in the study. For patients without schizophrenia spectrum conditions (SSC), the inclusion criteria were for them to have any other diagnosis except for schizophrenia spectrum conditions, be of legal age (>18 years of age), and have no medical comorbidities. Exclusion criteria were the same as those for subjects. The study data were collected by two psychiatrist volunteers (PF and SK) who were appropriately trained in test administrations by the principal investigator (LL). The administration of the test took approximately 1 hour to 1 hour and 30 minutes per patient. The flow of the administration was as follows: patients were administered the Pictogram Test (PT) encoding part; then in an hour between encoding and retrieval, they were administered the PANSS-N5 and Mood Differentiation Questionnaire (MDQ), followed by the retrieval part of the PT. The testing took place in a private room, free from distractions and noise.

The new test battery can be easily implemented in the clinical setting. The education and training on the administration and scoring of the PANSS-N5 and the PT takes 30 minutes. Social workers, psychologists, psychiatry residents, medical students, psychiatrists, psychiatric registered nurses, and psychiatric nurse practitioners and physician assistants can be trained in the administration and scoring of the battery. The data from the battery will aid the psychiatric providers who diagnose patients suspected of having schizophrenia spectrum illnesses in a more accurate diagnosis and treatment plan. 

The PANSS-N5

The Abstract Thinking subscale (N5) of the PANSS consists of 32 items: 16 Similarities and 16 Proverbs. Both Similarities and Proverbs are divided into blocks of four, with easier items at the beginning and more difficult items toward the end. After the explanation of the test, the examiner reads the Similarities one by one and records the examinee’s answer verbatim. The same goes for Proverbs. The scoring is done after the examinee’s answers are recorded.

Scoring

Each of the four item blocks of Similarities and Proverbs is given an average score, which consists of each item scoring from 1, 2, 3, or 4. A score of 1 represents a correct/adequate response, 2 a marginal but coherent response, 3 a functional/concrete response, and 4 a significant difficulty in abstraction. The total score for Similarities is the sum of the averages of all four blocks of Similarities words. Thus, the ideal, correct sum was 4; the incorrect sum was 16. The same scoring applied to Proverbs.

Interpretation

The ideal, correct sum was 4; the incorrect sum was 16. Scores between 4 and 16 represent the degree of impairment in abstract thinking, with higher numbers indicating more difficulty in abstraction.

The Pictogram Test

The detailed description of this test is outlined elsewhere [17]. Briefly, the examiner is given orally 16 words and phrases (targets) one by one that go progressively more difficult in their level of abstraction. The examinee is to draw something after each target is announced that would help to recall the target. Artistic ability is not judged. The drawings are taken away at the end of the test and re-presented to the examinee after one hour for retrieval. During the retrieval, examinees are asked to recall the target and give a brief explanation about why they had constructed the drawings for each target. Thus, the drawings and explanations of how they related to the targets were considered in scoring.

The Mood Differentiation Questionnaire

The study’s questionnaire draws from Robert Powell’s book “Differentiation of Moods: As a Reflection of Ego Organization and Personality Style” [15].

Based on prior cognitive and symbolic processing studies in schizophrenia, a medium-to-large effect size was anticipated (Cohen’s d ≈ 0.8) for between-group differences on PT performance. Using a two-tailed independent samples t-test, an alpha level of 0.05, and a desired statistical power of 0.80, a total sample size of approximately 40 participants (20 per group) was required to detect such an effect.

Theory and calculation

The PANSS-N5 (which has robust validity) was scored by LL and DW to ensure inter-rater reliability. The PANSS-N5 was scored using the scoring guide described in detail in another article [7]. Briefly, each of the four item blocks of Similarities and Proverbs was given an average score, which consisted of each item scoring from 1, 2, 3, or 4. A score of 1 represents a correct/adequate response, 2 a marginal but coherent response, 3 a functional/concrete response, and 4 a significant difficulty in abstraction. The total score for Similarities was the sum of the averages of all four blocks of Similarities words. Thus, the ideal, correct sum was 4, and the incorrect sum was 16. The same scoring applied to Proverbs.

For the MDQ, the score for a specific question could range between 1 (normal/neurotic) and 4 (incorrect/psychotic response). Each of the five mood questions was scored separately and then aggregated to determine the final score for the subject. The inter-rater reliability of this questionnaire has been demonstrated in a previous research study.

The Mood Differentiation Questionnaire was scored by LL applying an improved scoring guide that was piloted in the previous study [15,16]. Briefly, each of the five questions was scored 1, 2, 3, or 4, with 1 being normal/neurotic, 2 being marginal but coherent, 3 being literal/functional demonstrating poorly differentiated mood, and 4 being an incorrect/psychotic response demonstrating undifferentiated mood.

The PT was scored by LL. For this analysis, we used variables that were found significantly contributory to discrimination between patients with schizophrenia spectrum conditions and normal controls in previous research [17,18]. Specifically, these variables were productivity of memorization, Attribute Index (AI), and Geometrical Index (GI).

Statistical analysis

A violin plot was used to visually display the distribution of each test score as a combination of a boxplot and a mirrored density plot. For each test score, we provided the summary statistics for patients with schizophrenia spectrum conditions and patients without the condition, respectively. The differences between the two groups were tested by two-sample t-tests.

Receiver operating characteristics (ROC) analyses were performed to evaluate the performance of the potential classification factors/models by calculating the sensitivities (true positive rates) and specificities (true negative rates) at each classification threshold. The area under the ROC curve (AUC) was present as an overall measure of the classification accuracy, and a bootstrap-based test was performed to assess whether the AUC was different from 0.5, the AUC of a non-informative classification model.

Furthermore, the optimal threshold was chosen to maximize the sum of sensitivity and specificity, and the p-value threshold used to determine significance was 0.05. Both sensitivity and specificity, given the optimal threshold, were calculated, together with the 95% confidence interval (CI) derived from bootstrap resampling. All factors with significant differences between the two groups were included in multiple logistic regression to derive a composite score for classification, which was essentially a weighted average of individual factors. The LASSO penalty was used as a penalty term of model complexity to handle correlations between test scores. The differences between different logistic models were tested by Rao’s score test.

All statistical analyses were conducted using R statistical software (R Foundation for Statistical Computing, Vienna, Austria).

## Results

The demographic and clinical characteristics of the study participants are summarized in Table 1. Briefly, the sample was predominantly Caucasian, with a mean age in the 30-40-year range. There were slightly more men than women, and most participants were housed, were unemployed, and had completed approximately 12 years of education, with no reported history of special education, who were stabilized on medications and responding well to medications, with an average duration of illness being 4-8 years. Participants in the schizophrenia spectrum group had been followed by psychiatrists for a longer duration and had a greater number of prior psychiatric hospitalizations compared to participants without schizophrenia spectrum conditions.

**Table 1:** Demographic and Clinical Characteristics of the Study Participants

Characteristic	Patients with schizophrenia (n = 20) (number (%))	Patients without schizophrenia (n = 20) (number (%))
Sex		
Male	16 (80%)	12 (60%)
Female	4 (20%)	8 (40%)
Race		
Caucasian	12 (60%)	16 (80%)
African American	5 (25%)	2 (10%)
Other	3 (15%)	2 (10%)
Living arrangement		
Housed	16 (80%)	18 (90%)
Homeless	4 (20%)	2 (10%)
Employment status		
Employed	2 (10%)	10 (50%)
Unemployed	18 (90%)	10 (50%)
Disability status		
On disability	10 (50%)	4 (20%)
Not on disability	10 (50%)	16 (80%)
Special education history		
Yes	5 (25%)	5 (25%)
No	15 (75%)	15 (75%)
Primary diagnosis		
Schizophrenia spectrum disorder	20 (100%)	0 (0%)
Mood disorder	0 (0%)	17 (85%)
Substance use disorder	0 (0%)	3 (15%)
Age (years)	38.6 ± 14.9	32.5 ± 11.8
Education (years)	12.3 ± 1.9	12.8 ± 2.2
Length of seeing a psychiatrist (years)	20.3 ± 14.4	7.6 ± 8.2
Number of previous inpatient admissions	8.9 ± 9.0	4.9 ± 6.3

Univariate analyses were conducted for each test score to assess group differences between patients with schizophrenia spectrum conditions and those without such conditions. Descriptive statistics and ROC analyses are presented in the tables corresponding to each measure.

Descriptive statistics for total MDQ scores are presented in Table 2. There were no statistically significant differences between the schizophrenia spectrum and control groups (p = 0.774). ROC analysis demonstrated limited to modest discriminatory ability, with an AUC of 0.652 (95% CI: 0.478-0.819) (Table 3). Given the lack of statistically significant group differences and relatively modest classification performance, MDQ scores were not included in subsequent multivariable analyses.

**Table 2:** MDQ in Patients With and Without Schizophrenia Spectrum Disorders Values are reported as mean ± SD. Group comparisons were performed using a two-sample t-test. MDQ: Mood Differentiation Questionnaire, SD: standard deviation

Group (number)	Mean ± SD	p-value	T-statistic
Control (n = 20)	2.6 ± 1	0.774	-1.66
Schizophrenia (n = 20)	2.5 ± 1.2		

**Table 3:** ROC Analysis of MDQ Scores AUC: area under the curve, CI: confidence interval, MDQ: Mood Differentiation Questionnaire, ROC: receiver operating characteristic

Metric	Value
AUC (95% CI)	0.652 (0.478-0.819)
Optimal cutoff	5.625
Sensitivity (95% CI)	0.75 (0.55-0.95)
Specificity (95% CI)	0.55 (0.35-0.75)

Scores on the PANSS-N5 (Similarities) did not differ significantly between groups (mean ± SD: 7.0 ± 2.0 for the schizophrenia spectrum group versus 6.0 ± 1.8 for controls; p = 0.099; Table 4). ROC analysis demonstrated moderate discriminatory ability, with an AUC of 0.754 (95% CI: 0.585-0.91). At the optimal cutoff value of 11.75, sensitivity was 0.70 (95% CI: 0.50-0.90) and specificity was 0.86 (95% CI: 0.70-1.00) (Table 5).

**Table 4:** PANSS-N5 (Similarities): Descriptive Statistics Values are presented as mean ± SD. Group comparisons were performed using a two-sample t-test. PANSS-N5: Positive and Negative Syndrome Scale - Negative Subscale, SD: standard deviation

Group (number)	Mean ± SD	T-statistic	p-value	Statistical test
Control (n = 20)	6.0 ± 1.8	-1.66	0.099	Two-sample t-test
Schizophrenia (n = 20)	7.0 ± 2.0			

**Table 5:** ROC Analysis of PANSS-N5 (Similarities) AUC: area under the curve, CI: confidence interval, PANSS-N5: Positive and Negative Syndrome Scale - Negative Subscale, ROC: receiver operating characteristic

Metric	Value
AUC (95% CI)	0.754 (0.585-0.91)
Optimal cutoff	11.75
Sensitivity (95% CI)	0.7 (0.5-0.9)
Specificity (95% CI)	0.86 (0.7-1.0)

In contrast, scores on the PANSS-N5 (Proverbs) were significantly higher in patients with schizophrenia spectrum conditions compared to controls (11.8 ± 3.3 versus 8.7 ± 3.6; p = 0.008; Table 6). ROC analysis indicated good discrimination between groups, with an AUC of 0.681 (95% CI: 0.516-0.833). Using the optimal cutoff value of 0.3, sensitivity was 0.3 (95% CI: 0.1-0.5) and specificity was 0.9 (95% CI: 0.9-1.00) (Table 7).

**Table 6:** PANSS-N5 (Proverbs): Descriptive Statistics Values are presented as mean ± SD. Group comparisons were performed using a two-sample t-test. PANSS-N5: Positive and Negative Syndrome Scale - Negative Subscale, SD: standard deviation

Group (number)	Mean ± SD	T-statistic	p-value	Statistical test
Control (n = 20)	8.7 ± 3.6	-2.84	0.008	Two-sample t-test
Schizophrenia (n = 20)	11.8 ± 3.3			

**Table 7:** ROC Analysis of PANSS-N5 (Proverbs) AUC: area under the curve, CI: confidence interval, PANSS-N5: Positive and Negative Syndrome Scale - Negative Subscale, ROC: receiver operating characteristic

Metric	Value
AUC (95% CI)	0.681 (0.516-0.833)
Optimal cutoff	0.3
Sensitivity (95% CI)	0.3 (0.1-0.5)
Specificity (95% CI)	0.9 (0.9-1.0)

For the Pictogram Test Productivity of Memorization, patients with schizophrenia spectrum conditions demonstrated significantly lower scores than controls (5.7 ± 4.0 versus 9.2 ± 4.4; p = 0.012; Table 8). ROC analysis showed good discriminatory performance, with an AUC of 0.761 (95% CI: 0.588-0.910). At the optimal cutoff of 8.25, sensitivity was 0.90 (95% CI: 0.75-1.00), while specificity was 0.70 (95% CI: 0.50-0.90) (Table 9).

**Table 8:** Pictogram Test Productivity of Memorization: Descriptive Statistics Values are presented as mean ± SD. Group comparisons were performed using a two-sample t-test. SD: standard deviation

Group (number)	Mean ± SD	T-statistic	p-value	Statistical test
Control (n = 20)	9.2 ± 4.4	2.63	0.012	Two-sample t-test
Schizophrenia (n = 20)	5.7 ± 4.0			

**Table 9:** ROC Analysis of Pictogram Test Productivity of Memorization AUC: area under the curve, CI: confidence interval, ROC: receiver operating characteristic

Metric	Value
AUC (95% CI)	0.761 (0.588-0.91)
Optimal cutoff	8.25
Sensitivity (95% CI)	0.9 (0.75-1.0)
Specificity (95% CI)	0.7 (0.5-0.9)

Similarly, Pictogram Test Attribute Index (AI) scores were significantly lower in the schizophrenia spectrum group compared to controls (47.0 ± 17.6 versus 58.5 ± 16.8; p = 0.041; Table 10). ROC analysis yielded an AUC of 0.716 (95% CI: 0.546-0.875). At the optimal cutoff value of 60.25, sensitivity was 0.80 (95% CI: 0.60-0.95) and specificity was 0.75 (95% CI: 0.55-0.90) (Table 11).

**Table 10:** Pictogram Test Attribute Index: Descriptive Statistics Values are presented as mean ± SD. Group comparisons were performed using a two-sample t-test. SD: standard deviation

Group (number)	Mean ± SD	T-statistic	p-value	Statistical test
Control (n = 20)	58.5 ± 16.8	2.11	0.041	Two-sample t-test
Schizophrenia (n = 20)	47.0 ± 17.6			

**Table 11:** ROC Analysis of Pictogram Test Attribute Index AUC: area under the curve, CI: confidence interval, ROC: receiver operating characteristic

Metric	Value
AUC (95% CI)	0.716 (0.546-0.875)
Optimal cutoff	60.25
Sensitivity (95% CI)	0.8 (0.6-0.95)
Specificity (95% CI)	0.75 (0.55-0.9)

Scores on the Pictogram Test Geometric Index (GI) were significantly higher in patients with schizophrenia spectrum conditions than in controls (7.0 ± 7.4 versus 3.1 ± 2.6; p = 0.033; Table 12). ROC analysis demonstrated modest discriminatory ability, with an AUC of 0.681 (95% CI: 0.516-0.833). At the optimal cutoff value of 7.5, sensitivity was low (0.30; 95% CI: 0.10-0.50), whereas specificity was perfect (1.00; 95% CI: 1.00-1.00) (Table 13).

**Table 12:** Pictogram Test Geometric Index Descriptive Statistics Values are presented as mean ± SD. Group comparisons were performed using a two-sample t-test. SD: standard deviation

Group (number)	Mean ± SD	T-statistic	p-value	Statistical test
Control (n = 20)	3.1 ± 2.6	-2.22	0.033	Two-sample t-test
Schizophrenia (n = 20)	7.0 ± 7.4			

**Table 13:** ROC Analysis of Pictogram Test Geometric Index AUC: area under the curve, CI: confidence interval, ROC: receiver operating characteristic

Metric	Value
AUC (95% CI)	0.681 (0.516-0.833)
Optimal cutoff	7.5
Sensitivity (95% CI)	0.3 (0.1-0.5)
Specificity (95% CI)	1.0 (1.0-1.0)

**Figure 1:**
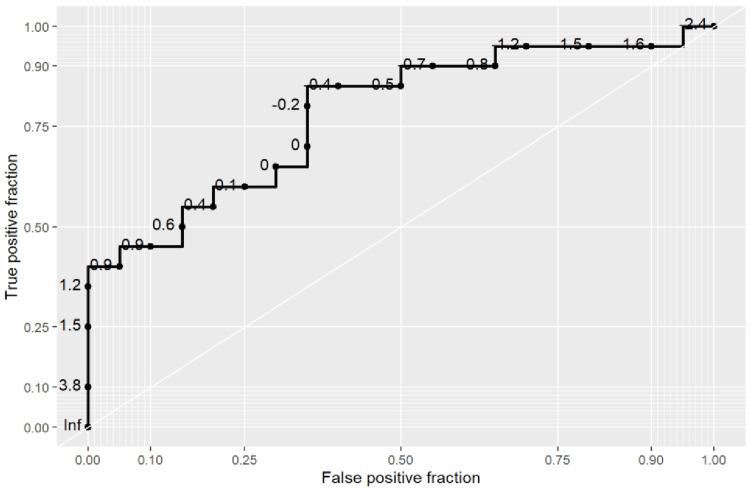
ROC for Composite Score From Logistic Regression Showing Significant Distinction in Patients With Schizophrenia and Patients Without Schizophrenia AUC = 0.782, 95% CI: 0.628-0.910 AUC: area under the curve, CI: confidence interval, ROC: receiver operating characteristic

Finally, a multivariate binomial logistic regression model incorporating PANSS-N5 Similarities, PANSS-N5 Proverbs, Pictogram Test Productivity of Memorization, Attribute Index, and Geometric Index demonstrated significant overall discrimination between schizophrenia spectrum and non-schizophrenia spectrum groups. Descriptive statistics for the composite score are presented in Table 14, with significantly higher scores observed in the schizophrenia spectrum group (p = 0.002). ROC analysis of the composite model yielded an AUC of 0.782 (95% CI: 0.628-0.910). At the optimal cutoff value of -0.4, sensitivity was 0.85 (95% CI: 0.70-1.00) and specificity was 0.65 (95% CI: 0.40-0.85) (Table 15 and Figure 1).

**Table 14:** Composite Logistic Regression Score: Descriptive Statistics Values are presented as mean ± SD. Group comparisons were performed using a two-sample t-test. SD: standard deviation

Group (number)	Mean ± SD	T-statistic	p-value	Statistical test
Control (n = 20)	-0.6 ± 1.0	-3.22	0.002	Two-sample t-test
Schizophrenia (n = 20)	0.7 ± 1.5			

**Table 15:** ROC Analysis of Composite Logistic Regression Model AUC: area under the curve, CI: confidence interval, ROC: receiver operating characteristic

Metric	Value
AUC (95% CI)	0.782 (0.628-0.91)
Optimal cutoff	-0.4
Sensitivity (95% CI)	0.85 (0.7-1.0)
Specificity (95% CI)	0.65 (0.4-0.85)

## Discussion

The results in our study are consistent with previous findings by Felsenheimer and Rapp (2024), which demonstrated that patients with psychosis experienced a decline in abstract scores and a rise in concrete scores for the Gorham Proverbs Test. These findings lend further credence to the hypothesis that patients with schizophrenia increasingly adopt concrete thinking due to their cognitive impairment, as well as to the study by Leontieva et al. (2022), which established construct validity of the Pictogram Test for the diagnosis of schizophrenia [9,18].

In Table 16, we see the comparison of the three tests’ administration time, scoring complexity, and discriminatory power.

**Table 16:** Comparison of the Three Tests’ Administration Time, Scoring Complexity, and Discriminatory Power AI: Attribute Index, AUC: area under the curve, GI: Geometric Index, MDQ: Mood Differentiation Questionnaire, PANSS-N5: Positive and Negative Syndrome Scale - Negative Subscale, PT: Pictogram Test

	PANSS-N5 (Proverbs)	PT	MDQ
Administration time	15 minutes	15 minutes	15 minutes
Scoring time	10 minutes	20 minutes	10 minutes
Discriminatory power: AUC	0.754	Memorization: 0.761, AI: 0.716, GI: 0.681	0.652 (non-significant)

Interpretation of findings

The PANNS-N5 Proverbs test results show that patients with schizophrenia scored significantly higher than patients without schizophrenia, highlighting poor abstract thinking in these patients [19]. The MDQ was not included in the final logistic regression analysis. We included PANSS-N5 Similarities despite the fact that it did not significantly discriminate between patients with schizophrenia spectrum disorders and without schizophrenia because it is part of the PANSS abstract thinking scale, and it gives a valuable estimate of patients’ premorbid intelligence level [20]. As predicted, the PANSS-N5 and the Pictogram Test demonstrated statistically significant differences between groups. However, given the limited sample size and pilot nature of the study, these findings should be interpreted as preliminary. While ROC analyses suggested moderate discriminatory ability, these estimates may be unstable and potentially overfit, particularly in the context of multivariable modeling with a small dataset. Therefore, the results should be viewed as hypothesis-generating rather than confirmatory. These findings suggest that these measures may contribute to the assessment of schizophrenia spectrum disorders, although they are not sufficient as standalone diagnostic tools.

Schizophrenia spectrum conditions are multifactorial in nature. To date, there are no brain scans that can definitely diagnose such complex, frequently difficult-to-treat, and at times devastating psychiatric disorder that consume resources [21], had suicide risk [22] and potential for violence [23], and has high disability rate [24], homelessness rate [25], substance abuse [26], and treatment cost, such as in the United States, which was $343.2 billion between 2013 and 2019 [27]. Being able to accurately diagnose schizophrenia condition may lead to early appropriate intervention, designing accurate help in the community, and a decrease in such devastating consequences as homelessness, polysubstance use, and even suicide [28].

The most significant findings are the following: we designed a short test battery that could be administered by any mental health professional after 30 minutes of education and training on administration and scoring. This test battery provides incremental validity for suspected diagnoses of schizophrenia spectrum disorders. Given the lack of psychologists working on inpatient psychiatric units (less that 30% of inpatient units have psychologists in the United States), this test battery can help determine an accurate diagnosis of schizophrenia spectrum disorder and design treatment and support accordingly [29].

Comparison with existing literature

These findings are consistent with prior studies examining abstract thinking deficits in schizophrenia. For example, Felsenheimer and Rapp (2024) [9] reported that patients with psychosis demonstrated decreased abstract reasoning and increased concrete responses on the Gorham Proverbs Test, supporting the shift toward concrete thinking in schizophrenia. Similarly, Leontieva et al. (2022) [18] established the construct validity of the Pictogram Test in diagnosing schizophrenia, highlighting its sensitivity to thought disorder [16,17].

The present study extends this literature by demonstrating that both proverb interpretation (PANSS-N5) and pictorial abstraction (PT) retain discriminatory power even when administered by non-specialist clinicians. This supports the broader applicability of these tools in real-world clinical settings, particularly where access to trained psychologists is limited.

Limitations

Several limitations should be considered when interpreting these findings. First, the sample size was small (n = 40), limiting statistical power and the stability of estimates derived from ROC analyses and multivariable modeling. As such, the reported classification performance may overestimate true diagnostic accuracy and requires replication in larger cohorts.

Second, the control group consisted of psychiatric patients rather than healthy individuals. While this enhances clinical relevance, it may also reduce the apparent discriminatory ability of the test battery and limit generalizability. Additionally, heterogeneity within the control group (e.g., mood disorders and substance use disorders) may have introduced variability that was not fully accounted for.

Third, feasibility was assessed primarily based on successful administration and subjective observations rather than objective metrics such as time-motion analysis, inter-rater reliability across multiple raters, or implementation outcomes. Therefore, conclusions regarding feasibility should be considered preliminary.

Finally, medication effects, cultural factors, and variability in examiner training may have influenced performance and were not systematically controlled.

Future directions

Future studies should include larger and more diverse samples, including healthy controls, to improve generalizability. Standardized and potentially digitalized scoring methods may enhance reliability and reduce administration burden. Further investigation of the MDQ is warranted, as prior studies suggest potential utility despite its lack of discrimination in this sample. Expanding the use of these tools in different clinical settings will help clarify their role in routine psychiatric assessment.

Clinical implications

This study demonstrates that brief cognitive assessments targeting abstract thinking can be feasibly administered by non-specialist providers with minimal training. Incorporating such tools into clinical practice may enhance diagnostic accuracy, improve characterization of thought disorder, and facilitate more targeted treatment planning for patients with schizophrenia spectrum disorders.

Although the study explored initial performance characteristics of the test battery, the use of the term “psychometrics” should be interpreted cautiously. Comprehensive psychometric validation, including assessments of reliability, construct validity, and external validity, was beyond the scope of this pilot study. Future research should systematically evaluate these properties in larger samples before drawing firm conclusions about the measurement characteristics of the battery.

## Conclusions

This pilot study explored the potential utility of a brief cognitive test battery in differentiating schizophrenia spectrum conditions from non-schizophrenia psychiatric controls. The Pictogram Test and PANSS-N5 demonstrated statistically significant group differences, whereas the Mood Differentiation Questionnaire did not. However, given the small sample size and exploratory nature of the analyses, including ROC and multivariable modeling, these findings should be interpreted with caution. The study provides preliminary evidence suggesting that elements of this battery may contribute to diagnostic assessment, but further validation in larger, more diverse samples is required.

Feasibility was supported by the successful administration of the battery by trained non-specialist clinicians, although a more objective and systematic evaluation of feasibility is needed. Future studies should focus on robust psychometric validation, inclusion of healthy control groups, and replication across different clinical settings, and could involve evaluating the cross-cultural diagnostic applicability of this battery across different cultures and languages beyond English.
